# The Immunoglobulin Superfamily Member Basigin Is Required for Complex Dendrite Formation in *Drosophila*

**DOI:** 10.3389/fncel.2021.739741

**Published:** 2021-11-04

**Authors:** Brikha R. Shrestha, Anita Burgos, Wesley B. Grueber

**Affiliations:** ^1^Department of Neuroscience, Columbia University Medical Center, New York, NY, United States; ^2^Department of Neuroscience, Zuckerman Mind Brain Behavior Institute, Columbia University, New York, NY, United States; ^3^Department of Physiology and Cellular Biophysics, Zuckerman Mind Brain Behavior Institute, Columbia University, New York, NY, United States

**Keywords:** Basigin, dendrite morphogenesis, *Drosophila*, dendritic arborization neurons, sensory neuron, dendrite-substrate interaction

## Abstract

Coordination of dendrite growth with changes in the surrounding substrate occurs widely in the nervous system and is vital for establishing and maintaining neural circuits. However, the molecular basis of this important developmental process remains poorly understood. To identify potential mediators of neuron-substrate interactions important for dendrite morphogenesis, we undertook an expression pattern-based screen in *Drosophila* larvae, which revealed many proteins with expression in dendritic arborization (da) sensory neurons and in neurons and their epidermal substrate. We found that reporters for Basigin, a cell surface molecule of the immunoglobulin (Ig) superfamily previously implicated in cell-cell and cell-substrate interactions, are expressed in da sensory neurons and epidermis. Loss of Basigin in da neurons led to defects in morphogenesis of the complex dendrites of class IV da neurons. Classes of sensory neurons with simpler branching patterns were unaffected by loss of Basigin. Structure-function analyses showed that a juxtamembrane KRR motif is critical for this function. Furthermore, knock down of Basigin in the epidermis led to defects in dendrite elaboration of class IV neurons, suggesting a non-autonomous role. Together, our findings support a role for Basigin in complex dendrite morphogenesis and interactions between dendrites and the adjacent epidermis.

## Introduction

Morphogenesis of neuronal dendritic arbors influences neuronal connectivity and functional specialization and is thus a critical step in nervous system development. Growing evidence indicates that dendrite morphogenesis is a tightly regulated process and that perturbations of the genetic programs that orchestrate it can result in defects that manifest both at the circuit and behavioral levels (Puram and Bonni, 2013; Dong et al., 2015). Unraveling the molecular basis of dendrite morphogenesis is therefore an important goal.

Neurons have complex cell-intrinsic molecular programs that regulate dendrite patterning and may be influenced by extrinsic factors (Corty et al., 2009; Dong et al., 2015). The interstitial spaces between neurons house a complex mélange of molecules secreted by diverse cell types that provide physical support as well as important developmental cues to neurons. In a growing nervous system, this rich extracellular molecular environment and the cellular substrates with which neurons interact change continuously in physical size and molecular profiles. For example, a developmentally programmed switch occurs in the composition of extracellular matrix (ECM) from an embryonic and early postnatal form to a mature adult form starting about 2 weeks after birth in the mammalian brain (Zimmermann and Dours-Zimmermann, 2008). Proper formation and subsequent maintenance or refinement of dendritic arbors must therefore involve precise coordination of arbor morphogenesis with such changes in the cellular/molecular substratum of neurons. Given the tremendous diversities of neuronal subtypes and their substrate environments across the nervous system, the mechanisms underlying such coordinative processes are likely very complex.

Several studies have begun to shed light on the molecular and cellular bases of dendrite-substrate interactions. In *Drosophila* larval sensory neurons, coordination of dendrite arbor size with that of the overlying epithelial cells is mediated via regulation of epithelium-ECM and epithelium-dendrite interactions by the microRNA *bantam* (Parrish et al., 2009; Jiang et al., 2014). Sensory neuron-ECM interactions mediated by integrins promote dendrite self-avoidance and maintenance by restricting branches largely to a two-dimensional plane (Han et al., 2012; Kim et al., 2012). Likewise, a ligand-receptor complex consisting of DMA-1 in neurons and SAX-7, LECT-2, and MNR-1 in the surrounding hypodermal tissue patterns the dendritic arbors of PVD mechanosensory neurons in *Caenorhabditis elegans* (Dong et al., 2013; Salzberg et al., 2013; Ziegenfuss and Grueber, 2013; Zou et al., 2016). Thus, adhesion receptors are strong candidates for providing signaling and attachment cues that promote dendritic elaboration, spatial patterning, and maintenance.

In this study, we sought to identify membrane-derived cues that promote dendritic elaboration, focusing on *Drosophila* larval dendritic arborization (da) sensory neurons. Following an expression pattern-based screen of publicly available protein-trap lines (Kelso et al., 2004; Buszczak et al., 2007), we focused on Basigin, an immunoglobulin (Ig) superfamily (IgSF) member and mediator of ECM remodeling in vertebrates. Despite its wide expression in the vertebrate brain (Allen Mouse Brain Atlas), the function of Basigin in the nervous system remains poorly understood. Basigin mediates cell-cell interactions between pre- and post-synaptic surfaces at the *Drosophila* neuromuscular junction (NMJ) (Besse et al., 2007), and between neurons and glia in the visual system (Fadool and Linser, 1993; Curtin et al., 2007). A recent study of the RNA binding protein Found in neurons (Fne) identified Bsg as one target that mediates sensory dendrite morphogenesis in neurons and substrate (Alizzi et al., 2020). These reports, together with the observed expression pattern, suggested that Basigin plays an important role in mediating neuron-substrate interactions that regulate dendrite morphogenesis. Our results confirm a cell-autonomous role for Basigin in neurons and also support a non-autonomous requirement in epidermal cells for proper dendrite morphogenesis. Structure-function analysis provided additional insights into Basigin function. We propose that Basigin mediates interactions between dendrites and epidermal cells that regulate dendrite morphogenesis in part by modulating the neuronal cytoskeleton through a conserved motif in its intracellular tail. Our findings also demonstrate the utility of an expression-based screen in identifying molecules that mediate dendrite morphogenesis.

## Materials and Methods

### Fly Genetics, Stocks and Reagents

Protein-trap lines utilized in our screen were made and provided by the laboratories of Dr. Allan Spradling (Carnegie Institution for Science) (Buszczak et al., 2007) and Dr. Lynn Cooley (Yale University) (Quiñones-Coello et al., 2007). The Basigin null allele *bsg^δ265^* (Curtin et al., 2007) was provided by Dr. Kathryn Curtin (University of Arkansas). Transgenic lines for expression of full-length Basigin (*UAS-bsg^*FL*^, UAS-bsg^*FL*^::GFP*) and mutant variants (*UAS-bsg^KRR > NGG^::GFP, UAS-bsg^extra^::GFP*) (Besse et al., 2007) were provided by Dr. Anne Ephrussi (EMBL Heidelberg). The Basigin RNAi line was obtained from the Vienna *Drosophila* RNAi Center (Transformant ID: 105293) (Dietzl et al., 2007). *IT(0871-Gal4)*, referred to as *871-Gal4* in the text, was provided by Dr. Thomas Clandinin (Stanford University). To generate the *UAS-Bsg-G* transgenic line, full-length cDNA of Bsg-G was first made by appending missing sequences to the partial Bsg-G cDNA obtained from the GH21853 cDNA clone (*Drosophila* Gold Collection, Berkeley *Drosophila* Genome Project). A c-terminal FLAG tag was added to the full-length Bsg-G cDNA and sub-cloned into a pUASTattB vector containing the *mini-white* gene. Plasmids were then injected into *Drosophila* embryos and transformants were selected based on eye color of adults. All other reagents were obtained from the Bloomington *Drosophila* Stock Center (Indiana University). Animals of either sex were used. Genotypes of animals analyzed for the experiments described herein are listed below. Animals were raised at 25°C, except those used for RNAi-based knockdown experiments, which were raised at 29°C.

### Mosaic Analysis With a Repressible Cell Marker Experiments

**Control:** *hsflp, GAL4*^*elav*^, *UAS-mCD8GFP*/+; *TubP-Gal80 FRT40A/FRT40A*

***bsg***^δ265^: *hsflp, GAL4^*elav*^, UAS-mCD8GFP/+; TubP-Gal80 FRT40A / bsg^δ265^ FRT40A*.

### Structure-Function/Rescue Experiments

**Control:** *hsflp, GAL4^*elav*^, UAS-mCD8GFP/+; TubP-Gal80 FRT40A / FRT40A*

***bsg***^δ265^: *hsflp, GAL4^*elav*^, UAS-mCD8GFP/+; TubP-Gal80 FRT40A / bsg^δ265^ FRT40A*

**Full-length Basigin**: *hsflp, GAL4^*elav*^, UAS-mCD8GFP/+; TubP-Gal80 FRT40A / bsg^δ265^ FRT40A; UAS-bsg^*FL*^ / +*

**Extracellular Basigin:** *hsflp, GAL4^*elav*^, UAS-mCD8GFP/+; TubP-Gal80 FRT40A / bsg^δ265^ FRT40A; UAS-bsg^*extra::GFP*^ / +*

**Basigin with KRR > NGG mutation**: *hsflp, GAL4^*elav*^, UAS-mCD8GFP/+; TubP-Gal80 FRT40A / bsg^δ265^ FRT40A; UAS-bsg^*KRR* > *NGG::GFP*^ / +*

**Dendritic localizations of full-length and mutated Basigin**: *ppk-Gal4/+; UAS-bsg^*FL::GFP*^* / +, *ppk-Gal4/+; UAS-bsg^*KRR* > *NGG::GFP*^/ +*, *ppk-Gal4 / bsg^δ265^ FRT40A; UAS-bsg^*KRR* > *NGG::GFP*^/ +*.

### Epidermal Basigin Knock Down and Overexpression

**Validation of IT (871)-Gal4 line**: *871-Gal4/UAS-mCD8GFP*

**Control:** *871-Gal4 / ppk-CD4tdGFP*

**Basigin-RNAi**: *UAS-bsg^*RNAi*^ / bsg^δ265^; 871-Gal4 / ppk-CD4tdGFP*

**Basigin overexpression**: *871-Gal4/UAS-Bsg-G*.

### Antibodies

The following antibodies were used: chicken anti-GFP (ab13970, Abcam, RRID:AB_300798, 1:500), goat anti-HRP (Sigma, 1:250), mouse anti-Coracle (c556.9 and c615.16, RRID:AB_1161644; developed by R. Fehon, 1:40), mouse anti-E-Cadherin (5D3, RRID:AB_528116; developed by B. Gumbiner, 1:100), rabbit anti-dsRed (632496, Clontech, RRID:AB_10013483:, 1:250), rat anti-Basigin (a kind gift from Anne Ephrussi (Besse et al., 2007), 1:100), rabbit anti-FLAG (Sigma, 1:100) and mouse anti-βPS integrin (CF.6G11, RRID:AB_528310; developed by D. Bower, 1:10). CF.6G11, 5D3, c556.9 and c615.16 were obtained from the Developmental Studies Hybridoma Bank developed under the auspices of the NICHD and maintained by the University of Iowa, Department of Biology. Species-specific secondary antibodies (Jackson Immunoresearch) raised in donkey were used at 1:250. Permeabilization was done with 0.3% Triton-X100 except for rat anti-Basigin staining which required Tween-20.

### Immunohistochemistry

Filleted 3^*rd*^ or 2^*nd*^ instar larvae were fixed in 4% paraformaldehyde for 18 min at room temperature on a tabletop shaker and stained using standard immunohistochemical techniques largely as described before (Grueber et al., 2002). Live-staining of Basigin was conducted by directly applying the primary antibody solution to filleted larvae and incubating for 15 min at room temperature. The animals were then rinsed thrice with 1x PBS every 3 min and fixed immediately using 4% paraformaldehyde. Subsequent staining procedures were identical to those used for fixed larval fillets.

### Quantitative Analysis

Morphometric analysis was conducted by tracing dendritic arbors using Neurolucida (RRID:SCR_001775, MBF Bioscience, United States). Dendrite tracing was carried out in confocal stacks captured using identical z-sectioning parameters (class IV) or flattened projections (class I). For Basigin rescue and epidermal knock-down experiments, the dorsal posterior quadrant was selected and quantified as a representative of the entire class IV dendritic tree. Validity of this approach was ascertained by comparing branches between the dorsal posterior quadrant and full arbor for each neuron in the control (*N* = 6) and *basigin* mutant (*N* = 9) groups. As shown in Supplementary Figure 2, the number of nodes in the posterior dorsal quadrant of each neuron constituted an equivalent proportion of, and scaled down linearly relative to, those over the entire arbor in both control and mutant neurons (proportions: 27.67 ± 3.40% and 26.81 ± 4.90%, respectively; *p* = 0.696; Supplementary Figures 2C,D). Furthermore, the extent of decrease in branching in *basigin* mutant neurons relative to the control was almost identical when quantified over the full arbor (26.71%) or only in the dorsal posterior quadrant (28.17%) (Supplementary Figure 2E).

Dendrite field size of class IV neurons was measured as the area of the smallest convex polygon enclosing the full dendritic tree (Convex Hull method) using the Hull and Circle plug-in (Karperien, 2005) in ImageJ (RRID:SCR_003070). In order to determine differences in the extent to which each arbor filled its dendritic field, coverage density (CD) was calculated as follows: a flattened confocal or traced image of a class IV dendritic tree was overlaid with a grid of square boxes (Supplementary Figure 2B). The size of the square box depended on the overall dimension of the arbor such that the box area was equal to the square root of area of the rectangle that wholly contained the dendritic tree. CD was then calculated as the ratio of number of boxes containing dendrites to the total number of boxes, multiplied by 100. The Box Counting feature in FracLac plugin (Karperien, 2013) for ImageJ was used wherever possible (traced images) for automated detection of boxes containing dendrites (Supplementary Figure 2B); others (confocal images) were analyzed manually with the genotype of each neuron masked prior to analysis.


$$\mathrm{CD}=\frac{N\,o.o\,f\,b\,o\,x\,e\,s\,c\,o\,n\,t\,a\,i\,n\,i\,n\,g\,d\,e\,n\,d\,r\,i\,t\,e\,s}{T\,o\,t\,a\,l\,n\,o.o\,f\,b\,o\,x\,e\,s\,i\,n\,t\,h\,e\,d\,e\,n\,d\,r\,i\,t\,i\,c\,f\,i\,e\,l\,d}\times 100$$

### Statistical Analysis

Statistical analysis was conducted using the R software package (R Project for Statistical Computing, RRID:SCR_001905). All data were checked for Gaussian distribution (Shapiro-Wilk test) and analyzed further by either Welch’s *t*-test or Wilcoxon Rank Sum Test, as appropriate, for two-sample comparisons. Multiple sample comparisons were done by performing Analysis of Variance (ANOVA) followed by a suitable *post hoc* test for pairwise comparisons as noted in figure legends. Statistical significance was inferred if *p* < 0.05. Data are presented as box plots in which the top and bottom box boundaries demarcate interquartile range (IQR) while whiskers represent 75^*th*^ percentile + 1.5^∗^IQR and 25^*th*^ percentile – 1.5^∗^IQR. Thick horizontal lines and black dots within boxes represent median and mean, respectively. Raw data are shown as points laid over box plots; placement of such points along the x axis was randomized within the constraints of group boundary to avoid visual occlusion when y values are similar.

## Results

### A GFP Trap Screen for Proteins Expressed in the Peripheral Nervous System and Nearby Cells

**FIGURE 1:**
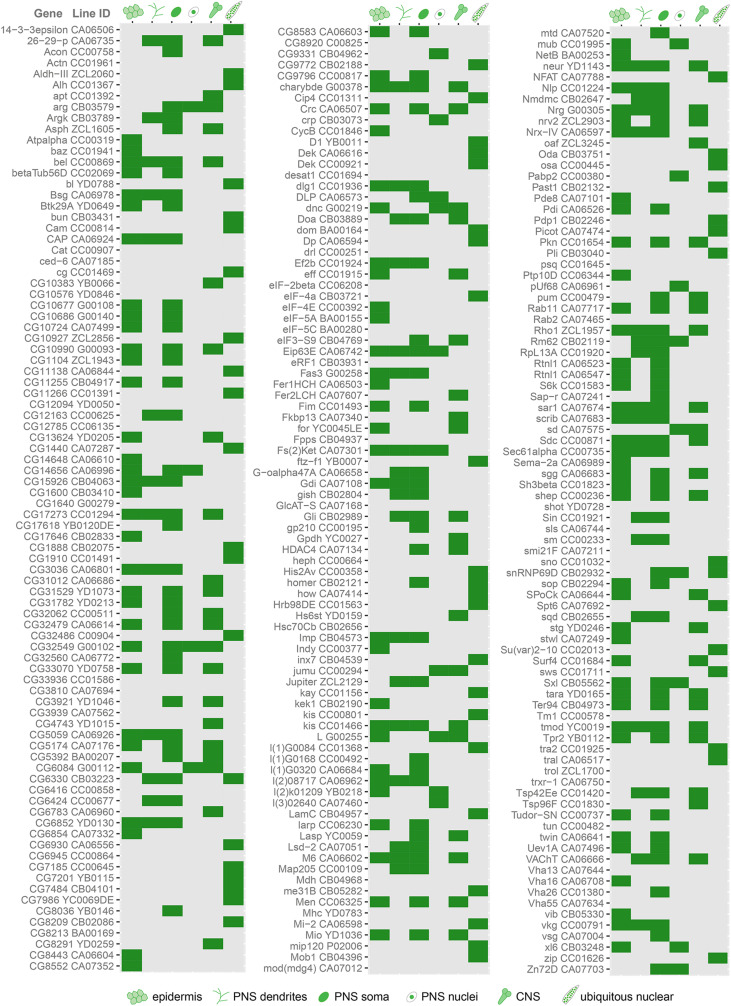
Cell type-specific protein expression and localization patterns in da neurons revealed by screen of protein trap lines. Chart showing the results of a screen of protein trap lines. Green indicates that GFP expression was observed, while gray indicates absence of detectable GFP signal by epifluorescence microscopy. Line ID refers to names assigned by creators of the lines, and the gene associated with each line is based on insertion site of the GFP-coding exon. Some lines exhibit ubiquitous nuclear GFP expression, which may be due to enhancer trapping and may not reflect true expression pattern of the associated gene (see text).

**FIGURE 2:**
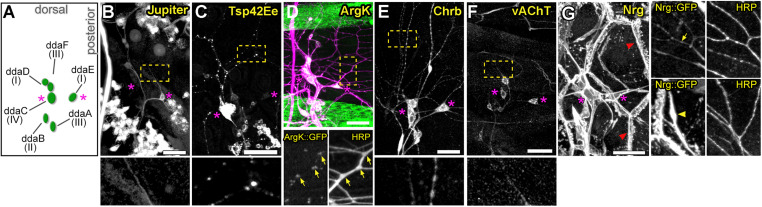
Expression patterns of select protein trap lines in dorsal cluster sensory neurons. **(A)** Schematic of typical cell body positions of da neurons in the dorsal cluster of the larval body wall. Labels show names and neuron class. Magenta asterisks in all panels mark positions of ddaE (right) and ddaC (left) for spatial reference. Examples of class-specific expression in Jupiter::GFP **(B)** and Tsp42Ee::GFP **(C)** lines, punctate dendritic GFP pattern in ArgK::GFP **(D)**, widespread sensory expression in Chrb::GFP **(E)** and vAChT::GFP **(F)** lines. Bottom panels show enlarged regions marked by yellow rectangles in panels above. Note correlation between ArgK::GFP localization and dendritic branch points of class I neuron ddaE (**D**, yellow arrows) **(G)** Example of a protein trap line (Nrg) with GFP expression in both neurons and epidermal cells. In the epidermis, strong localization is seen at cell borders (red arrowhead). Panels on the right are close-ups showing regions with strong localization in neuronal dendrites (top panel, yellow arrow), and in epidermal cells (bottom panel, yellow arrowheads) underneath dendrites. Scale bars, main panels: 25 μm **(B-G)**, enlarged bottom or side panels: 75 μm **(B,C,E,F)**, 65 μm **(D)** and 50 μm **(G)**.

**FIGURE 3:**
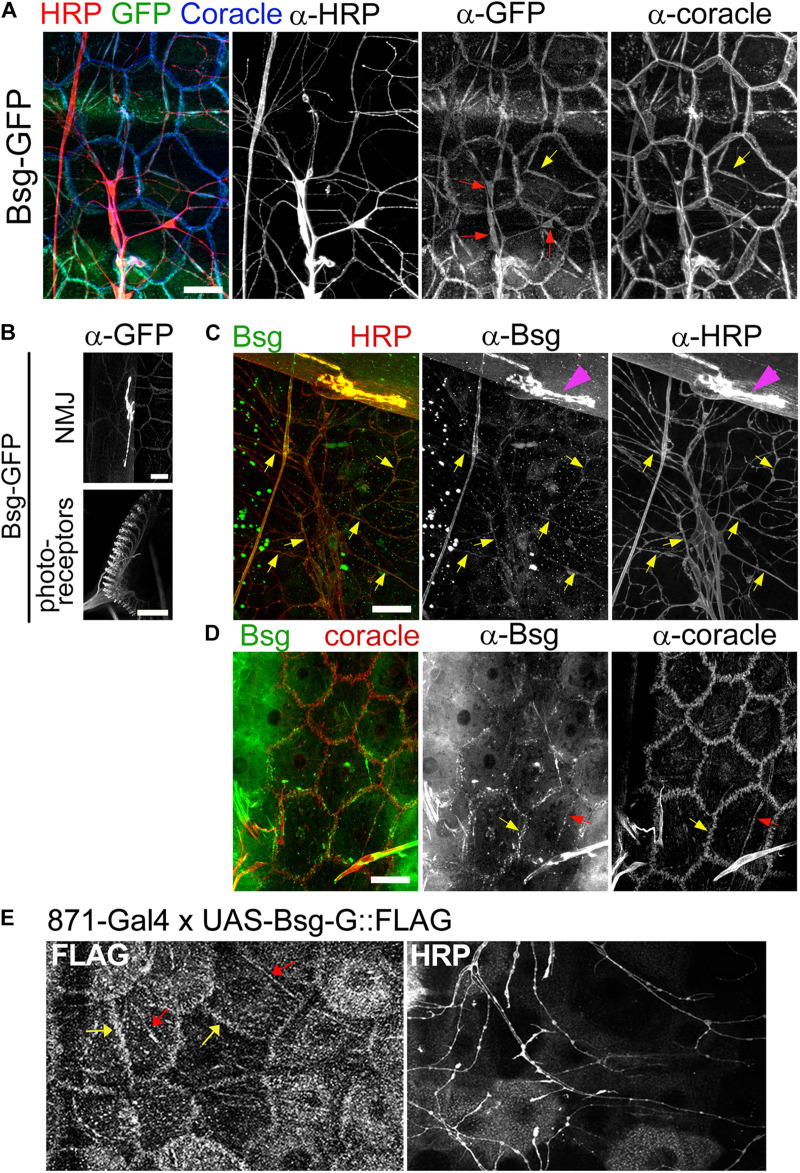
Basigin is expressed in da neurons and their epidermal cell substrates. **(A)** Basigin::GFP expression in epidermal cells and da neurons in the *Drosophila* larval body wall at third instar stage. Basigin::GFP localized to soma (red arrows), axons and dendrites of neurons. In the epidermis, Basigin::GFP localized to cell borders and along dendrite segments (yellow arrows), overlapping almost completely with the septate junction resident protein Coracle. **(B)** Basigin::GFP in the larval NMJ and photoreceptors, two structures that have previously been shown to require Basigin for proper morphogenesis (Besse et al., 2007; Curtin et al., 2007). **(C)** *w*^1118^ larvae were labeled with α-Bsg and α-HRP under standard immunohistochemical conditions. Basigin expression was observed in da neurons, with localization to dendrites (yellow arrows) and axons, and also in NMJ (magenta arrowheads), but epidermal expression was not detected. **(D)** When α-Bsg was applied to live tissue before fixing, Basigin was detected at cell borders (yellow arrows) and along dendrite segments that overlapped with α-Coracle staining (red arrows). **(E)** Expression of FLAG-tagged Basigin (UAS-Bsg-G) in the epidermis and staining with α-FLAG (left panel) and α-HRP (right panel) revealed Bsg-G localization at epidermal cell borders (yellow arrows) and tracking dendrite segments (red arrows), which is consistent with the observed epidermal GFP pattern of Basigin-GFP protein-trap lines. Scale bars, 25 μm.

To identify genes involved in dendrite morphogenesis, we examined the expression patterns of >250 genes in third instar *Drosophila* larvae using protein trap insertion lines generated previously (Morin et al., 2001; Buszczak et al., 2007; Quiñones-Coello et al., 2007). Each of the screened lines contained a GFP-coding exon inserted in a gene locus, which results in GFP-tagging of its protein products. Larval peripheral nervous system (PNS) expression data for all protein traps that we examined is provided in Figure 1. Approximately half of the lines showed expression in the larval PNS (Figures 1, 2). Of those with predominantly neuronal expression, some showed different class-specific levels of expression in da neurons. For instance, Jupiter::GFP showed strong expression in class I da neurons (Figure 2B). We observed punctate localization along dendrites in several lines (e.g., Tsp42Ee::GFP, ArgK::GFP, and Chrb: GFP) (Figures 2C–E). On the other hand, VAChT::GFP was expressed in all neurons, but at discrete levels anti-correlated with the branching complexity of da neurons—high in class I and low in class III and IV neurons (Figure 2F). A subset of lines showed ubiquitous nuclear expression (e.g., CB04957 for LamC, Figure 1) that may not reflect bona fide protein expression and localization due to enhancer trapping, as explained previously (Buszczak et al., 2007). However, some lines showed variable or non-nuclear GFP expression in addition to ubiquitous nuclear GFP (e.g., CB02121 for homer, Figure 1). A total of 78 lines showed expression in both neurons and epidermis. A subset of these lines featured dendritic GFP localization in neurons, and strong but intermittent epidermal enrichment of GFP adjacent to dendrites (e.g., Nrg::GFP in Figure 2G). We chose to focus further on the multifunctional immunoglobulin superfamily member Basigin (Bsg) based on its expression in both neurons and epidermal cells (Figure 3A).

### Basigin::GFP Fusions Show Expression in da Neurons and Epidermal Substrate

Bsg-GFP (Line ID: CA06978) contains a GFP exon cassette inserted in an intron in the *basigin* locus. Staining of filleted Bsg-GFP third instar larvae with anti-GFP revealed signal in diverse tissues. Strong expression was observed in the larval photoreceptors and NMJ (Figure 3B), both of which are known to require Basigin for proper development (Besse et al., 2007; Curtin et al., 2007). In the larval PNS, we observed Bsg::GFP in da neuron cell bodies, axons, and dendrites (Figure 3A). Additionally, we observed expression in epidermal cells that lie in close proximity to da neuron dendrites. The sub-cellular localization of GFP in the epidermal cells overlapped with that of Coracle (Figure 3A), a septate junction resident protein that marks regions where da neuron dendrites become enclosed within epidermal cell invaginations (Kim et al., 2012). We examined GFP expression in two other independently generated protein-trap lines (Bsg-GFP-2 and Bsg-GFP-3) that harbor GFP-coding exon insertions at different sites within the *basigin* locus. Both lines showed similar expression in neurons and epidermis as described above (Supplementary Figures 1A,B). We additionally labeled wild-type larvae with Basigin antibody. Labeling was observed in da neurons, including along dendrites and also at the NMJ (Figure 3C). We did not observe specific epidermal localization in fixed tissue, however, labeling was apparent when the primary antibody was applied to unfixed preparations (Figure 3D). The reason for this discrepancy is not known, but it is conceivable that epidermal expression is masked by fixation, or that the expression pattern of the three Bsg-GFP traps is somehow aberrant. To examine epidermal localization independent of GFP tagging we generated a transgenic FLAG-tagged Basigin (UAS-Bsg-G) line and drove expression in epidermal cells using the InSITE line *{IT.GAL4}871* (Gohl et al., 2011). Specificity of the driver was verified using a fluorescent reporter line. Strong GFP signal was observed across the epidermis of *871-Gal4/+;UAS-mCD8::GFP/+* larvae (Supplementary Figures 1C,D) but none was detected in da neurons (Supplementary Figure 1D’), indicating the epidermal specificity of *871*-*Gal4* in larvae. Strong FLAG signal was observed along epidermal cell boundaries and along epidermis adjacent to dendrite branches (Figure 3E), similar to the Bsg::GFP localization patterns observed in the protein trap lines. Taken together, these data support expression of Basigin in da neurons and also in epidermal cells in the larval body wall, and localization of these sources of Basigin in adjacent intercellular regions.

### Basigin Is Required in Class IV da Neurons for Proper Dendritic Morphogenesis

**FIGURE 4:**
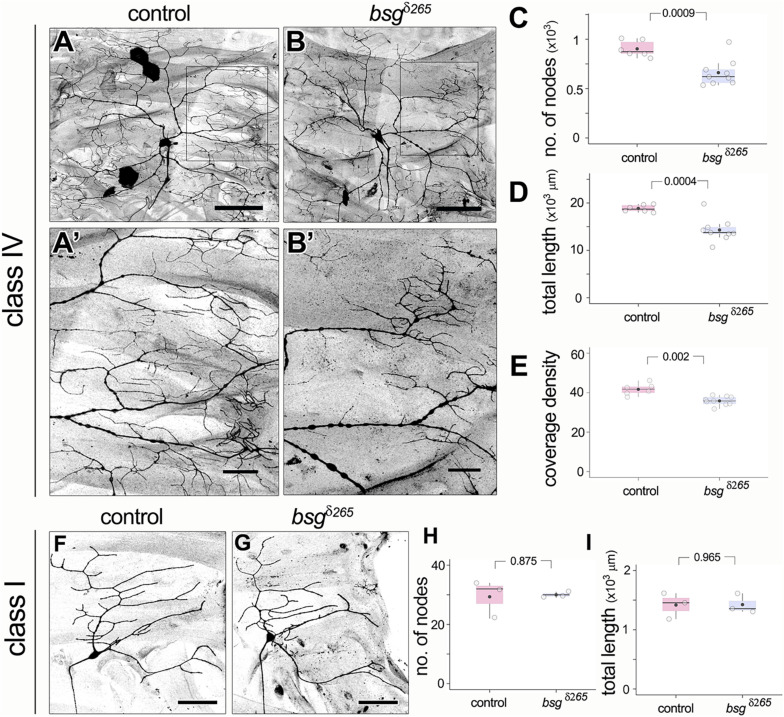
Basigin is required for morphogenesis of dendritic arbors. In contrast to control class IV neurons **(A,A’)**, *bsg^δ265^* class IV MARCM clones **(B,B’)** develop dendritic arbors with significantly fewer branches **(C)** and reduced total dendrite length **(D)** at late third instar stage. Insets **(A’)** and **(B’)** show magnified views of the boxes marked in **(A)** and **(B)**, respectively. Dendrite coverage density **(E)** of mutant neurons was significantly lower. In contrast, class I da neurons were unaffected by loss of Basigin. Comparison of class I control MARCM clones and *bsg^δ265^* neurons **(F,G)** revealed no difference in number of dendrite branch nodes **(H)** or total dendrite length **(I)**. Scale bar, 100 μm in **(A,B)**, 25 μm in **(A’,B’)** and 50 μm in **(F,G)**. *p* values are indicated for Welch’s *t*-test.

Based on Basigin expression in the PNS, we next investigated whether Basigin is involved in regulating neuronal morphogenesis in sensory neurons. We performed loss of function analysis using the Mosaic analysis with a repressible cell marker (MARCM) approach (Lee and Luo, 1999). We examined the null allele *bsg^δ265^* in which a part of the Basigin locus including the start codon is deleted (Curtin et al., 2005). Loss of Basigin in class IV neurons (ddaC) resulted in aberrant dendritic arbors in third instar stages. In contrast to the complex space-filling dendrites of control class IV neurons (Figure 4A), *bsg^–/–^* class IV neurons (Figure 4B) had significantly fewer branches (Control: 903 ± 82, *N* = 6; bsg^δ265^: 662 ± 136, *N* = 9; *p* = 0.0009) and reduced total dendrite length (Control: 18846 ± 751 mm, *N* = 6; bsg^δ265^: 14297 ± 2484 mm, *N* = 9; *p* = 0.0004) (Figures 4C,D). By contrast, there was no significant difference in dendritic field area between control and mutant neurons (Supplementary Figure 2A). These results suggest that mutant neurons are able to scale their dendritic territory to an appropriate size for the third instar stage and that the dendrite phenotype reflected a defect in space filling. Indeed, we found significantly lower dendrite coverage density (a dimensionless metric; see “Materials and Methods” section) in mutant neurons compared to control neurons (Control: 41.7 ± 2.9, *N* = 6; bsg^δ265^: 35.81 ± 2.21, *N* = 9; *p* = 0.002) (Figure 4E).

In contrast to class IV neurons, *bsg^–/–^* class I dendritic arbors were comparable to those of control class I neurons (Figures 4F,G) with statistically identical branch number (Control: 29 ± 6, *N* = 3; bsg^δ265^: 30 ± 1, *N* = 3; *p* = 0.875) and total dendrite length (Control: 1418 ± 219 mm, *N* = 3; bsg^δ265^: 1425 ± 165 mm, *N* = 3; *p* = 0.965) (Figures 4H,I). Thus, our data indicate that Basigin is cell-autonomously required for morphogenesis of complex space-filling dendritic arbors in da neurons.

### Loss of Basigin in Neurons Causes a Developmental Defect in Dendrite Elaboration

**FIGURE 5:**
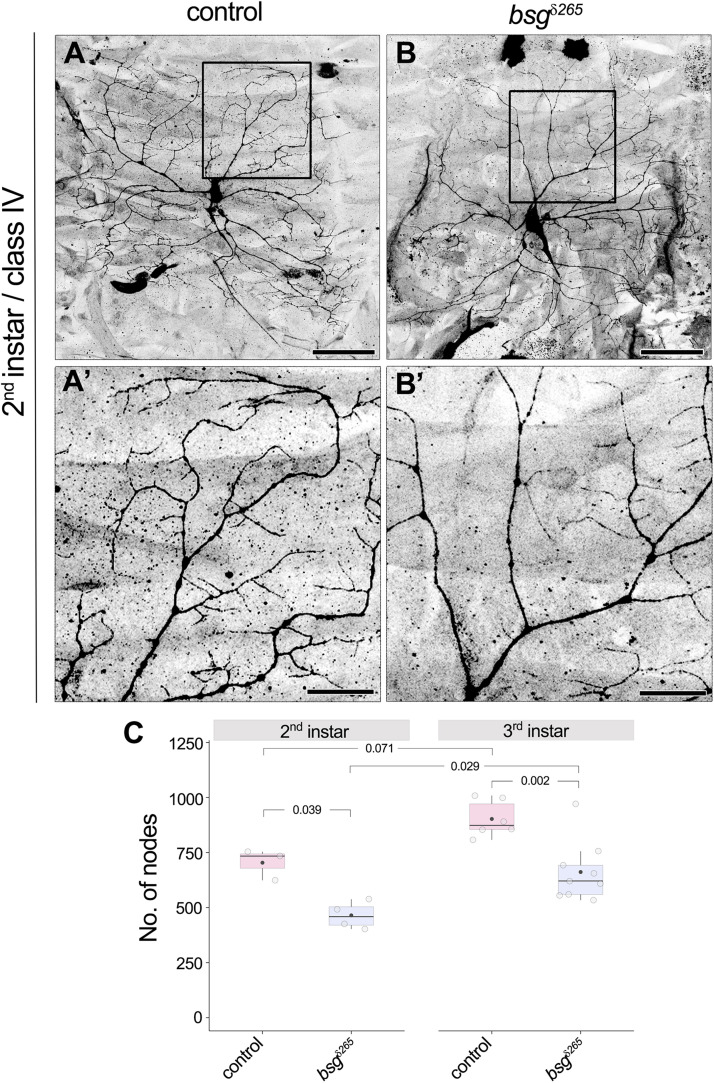
Developmental defect in branch elaboration contributes to formation of aberrant dendritic arbors in *bsg^–/–^* class IV neurons. Comparison of dendrites of control **(A,A’)** and *bsg^δ265^* class IV MARCM clones **(B,B’)** at late second instar stage revealed significantly fewer branches in the latter group of neurons **(C)**, indicating that inadequate branch elaboration during development contributes to the phenotype observed at late third instar stage. Insets **(A’)** and **(B’)** show magnified views of the boxes marked in **(A)** and **(B)**, respectively. The number of nodes in neurons of third instar larvae is also shown for comparison in **(C)**. As in control neurons, significant increase in branching occurred between the second and third instar stages in *bsg^–/–^* class IV neurons **(C)**, which indicates that dendrite growth and branching are not completely halted upon loss of Basigin. Scale bars, 50 μm in **(A,B)** and 20 μm in **(A’,B’)**. *p* values are indicated for Tukey’s HSD conducted following two-way ANOVA with genotype and developmental stage as independent categorical factors (*p* = 0.00007 and 0.00073, respectively).

*Drosophila* larvae show a drastic increase in body size from the first to third instar stages. Although class IV neurons establish their complete tiling pattern by the end of the first instar stage, they continue to elaborate branches to maintain full coverage of their territories as the animal grows (Parrish et al., 2009). Our observation of reduced dendrite branching and coverage density in *bsg^–/–^* neurons at late third instar in the absence of change in dendritic field size suggested a possible defect in addition of new branches during development. To determine if *bsg^–/–^* mutant class IV neurons have aberrant branch elaboration programs, we examined their dendrite morphology at 72 h after egg-laying (AEL), a stage that marks the end of second instar and is characterized by active dendrite elaboration. Consistent with previous reports (Parrish et al., 2009), we found that control class IV neurons have highly branched dendritic arbors that completely innervate their dendritic fields at this stage (Figures 5A,A’). In contrast, *bsg^–/–^* class IV neurons at the same stage had simpler dendrite arborization with ∼34% fewer branches (Figures 5B,B’) compared to control neurons (No. of nodes, Control: 704 ± 70, *N* = 3; bsg^δ265^: 465 ± 62, *N* = 4; *p* = 0.039). Dendrite arborization nevertheless continued to increase in *bsg^–/–^* neurons, with significant change from late second to late third instar (No. of nodes, 465 ± 62, *N* = 4 at 2^*nd*^ instar vs. 662 ± 136, *N* = 9 at 3^*rd*^ instar; *p* = 0.029), as in control neurons (No. of nodes, 704 ± 70, *N* = 3 at 2^*nd*^ instar vs. 903 ± 82, *N* = 6 at 3^*rd*^ instar; *p* = 0.071). The average number of branches added between late 2^*nd*^ and late 3^*rd*^ instars was nearly equivalent between *bsg^–/–^* and control neurons (197 and 199 branches, respectively), indicating that mutant neurons show no defects in branch addition at late larval stages (Figure 5C). Although these experiments do not rule out possible contribution of late-stage branch maintenance defects, our data are consistent with a primary defect in *bsg^–/–^* neurons throughout larval development, which, in wild-type animals, features prolific dendrite elaboration to keep up with the expanding body wall (Parrish et al., 2009).

### Membrane-Tethering and a Conserved Intracellular Motif of Basigin Are Required for Its Function in Neurons

**FIGURE 6:**
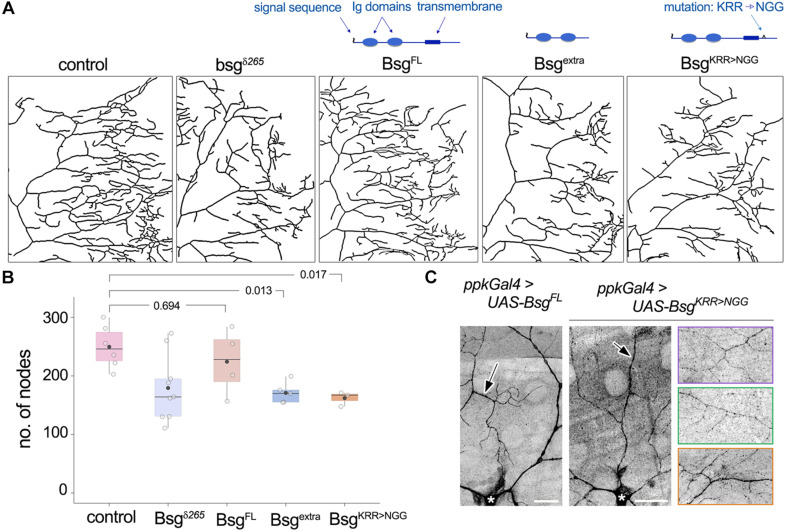
Structure-function analysis of Basigin in dendrite morphogenesis. **(A)** Dendrite traces of posterodorsal quadrants of class IV neurons under control, mutant, and various rescue conditions. Schematics indicate functional domains in full-length Basigin (Bsg^*FL*^), a truncated variant containing only the extracellular Ig domains (Bsg^extra^), and a mutant variant with the KRR motif in the intracellular region changed to NGG (Bsg^KRR > NGG^). Bsg^*FL*^ rescued the reduction in branching observed in *bsg^–/–^* MARCM clones of class IV neurons **(A,B)**. Neither Bsg^extra^ nor Bsg^KRR > NGG^ was able to rescue, indicating that membrane-tethering and integrity of the intracellular KRR motif are essential for Basigin function **(A,B)**. **(C)** GFP-tagged Bsg^*FL*^ and Bsg^KRR > NGG^ both showed strong localization to class IV dendrites when expressed under the *ppk-Gal4* driver. Arrows indicate GFP signal in dendrites and * indicates cell body of the class IV neuron ddaC. Rightmost images showcase localization of Bsg^KRR > NGG^::GFP to fine terminal branches of class IV neurons. Each colored panel is from a different animal. *p* values are indicated for Tukey’s HSD following one-way ANOVA (*p* = 0.0058) **(B)**. Scale bars, 25 μm.

Basigin is a single-pass transmembrane protein with two predicted Ig domains in its N-terminal extracellular region and a short intracellular C-terminal ending. Its transmembrane region is highly conserved and the juxtamembrane KRR motif, a putative binding site for cytoskeletal organizers (Yonemura et al., 1998), is important for regulation of NMJ morphology in flies (Besse et al., 2007). The N-terminal end of Basigin contains a signal sequence, suggesting that it may be released extracellularly in some form, likely upon cleavage of the full-length protein. Indeed, evidence from vertebrate studies indicates that Basigin is secreted in microvesicles by human uterine epithelial cells, and the secreted peptides induce MMP expression in human uterine fibroblast cells (Braundmeier et al., 2012). To determine the domain requirements for *Drosophila* Basigin during dendrite morphogenesis, we examined the ability of mutated variants of Basigin (Figure 6A) to rescue the dendrite morphogenesis defects described above. Full-length Basigin protein and variants with mutations or truncations in putative functional domains were expressed exclusively in *bsg^–/–^* cells by adding the respective transgenes into the crossing scheme used to generate *bsg^–/–^* MARCM clones. For quantification of dendritic branching we focused analysis on single quadrants of class IV arbors. We validated this method by examining the loss-of-function clones described above (Figure 4), and found that the number of nodes in the posterodorsal quadrant constituted equivalent proportions of nodes over the entire arbor in control and mutant animals (Supplementary Figure 2C). Furthermore, no significant difference was observed when the extent of reduction in branching in *bsg^–/–^* class IV neurons was quantified over the whole arbor or the posterodorsal quadrant (Supplementary Figure 2E). Together, these results indicated that the posterodorsal quadrant could be used as a proxy for branching across the arbor for these genotypes.

Expression of full-length wild-type Basigin (Bsg^*FL*^) rescued dendrite defects in *bsg^–/–^* class IV neurons (No. of nodes in posterior dorsal quadrant, Control: 249.67 ± 36.57, *N* = 6; bsg^δ265^: 179.33 ± 56.54, *N* = 9; Bsg^*FL*^: 224.25 ± 56.51, *N* = 4). By contrast, Basigin lacking its transmembrane and cytoplasmic regions (Bsg^extra^) failed to rescue dendritic branching defects in *bsg^–/–^* class IV neurons (Bsg^extra^: 171 ± 18.18, *N* = 5, Figures 6A,B). Likewise, full-length Basigin with point mutations that substituted the juxtamembrane KRR basic residues in the cytoplasmic tail to NGG (Bsg^KRR > NGG^) failed to rescue the branching defects of *bsg^–/–^* neurons (Bsg^KRR > NGG^: 162 ± 12.29, *N* = 3, Figures 6A,B). Since motifs in the cytoplasmic tail of cell surface proteins may be essential for proper sub-cellular localization, we examined if the lack of rescue by the mutant Basigin variant could be explained by a defect in proper subcellular localization. GFP-tagged full-length Basigin expressed under the class IV neuron-specific *ppk-Gal4* driver showed robust localization to dendrites (Figure 6C), consistent with data from the Bsg-GFP trap line as well as anti-Basigin staining of wild-type animals (Figure 3). Likewise, GFP-tagged Bsg^KRR > NGG^ showed stable expression in class IV neurons with strong localization to dendrites including fine terminal branches (Figure 6C). Therefore, gross mislocalization of Bsg^KRR > NGG^ proteins within neurons is unlikely to account for their inability to rescue dendrite elaboration defects. Our data do not eliminate the possibility that lack of rescue by Bsg^extra^ – despite its ability to partially rescue some NMJ phenotypes (Besse et al., 2007)– may be attributable to aberrant trafficking of the truncated protein. Taken together, our results indicate that the function of Basigin in regulating dendrite elaboration of class IV neurons requires membrane-tethering and an intact intracellular KRR motif.

### Non-autonomous Role for Basigin in Dendrite Morphogenesis

**FIGURE 7:**
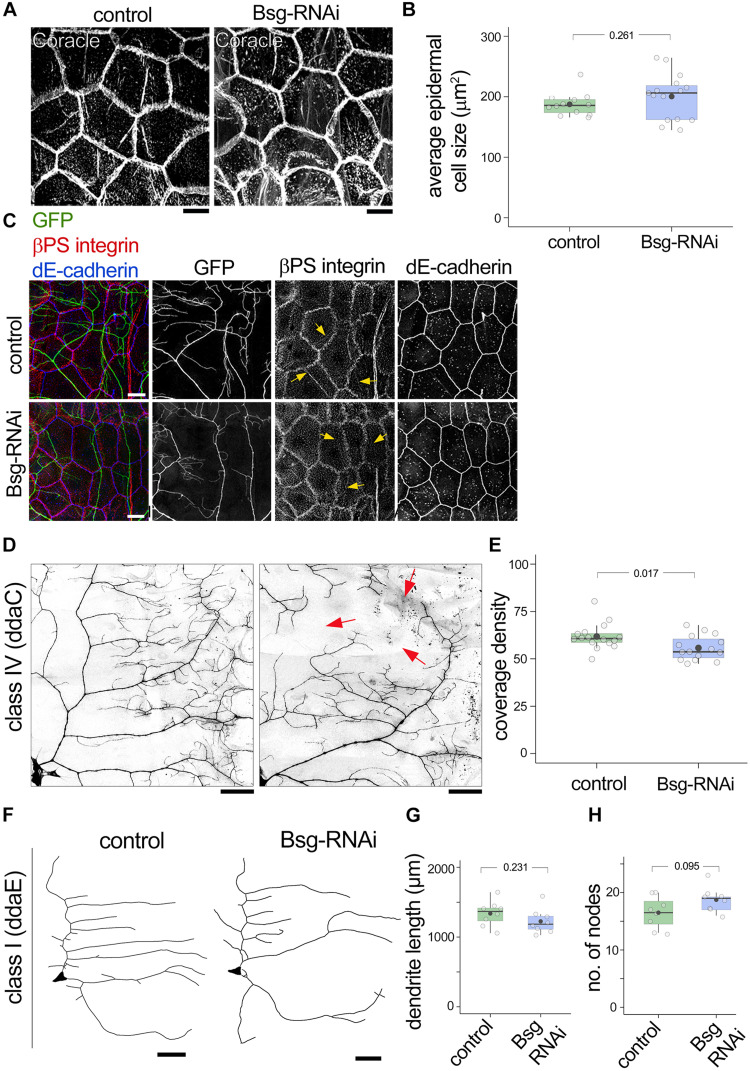
Basigin is required cell-non autonomously in substrate epidermal cells for class IV dendrite morphogenesis. **(A)** Basigin was knocked down in the epidermis by driving *UAS-bsg-RNAi* using the 871-Gal4 driver. Gross morphology of epidermal cells and expression of the septate junction resident protein Coracle remained unaltered **(A,B)**. Epidermal localization of βPS integrin and dE-cadherin **(C)** were indistinguishable in control and Bsg-RNAi animals. Notably, epidermal βPS integrin localization along dendrites (yellow arrows in **C**) persisted. GFP signal in **(C)** is from the *ppk-CD4tdGFP* transgene. **(D,E)** Knocking down Basigin in the epidermis caused aberrant dendrite morphogenesis in class IV da sensory neurons. Red arrows in **(D)** mark areas with large gaps in dendritic field. Class I neurons **(F)** did not exhibit significant change in total dendrite length **(G)** or number of nodes **(H)**. Scale bar, 25 μm. *p* values are indicated for Welch’s *t*-test.

Given our evidence for Basigin expression in epidermal cells and their close association with da neuron dendrites, we next examined possible cell non-autonomous roles for Basigin in regulating dendrite morphogenesis. For this experiment, we used the *871-Gal4* line (Supplementary Figures 1C,D) to drive *UAS-bsgRNAi* in the epidermis. Our results showed that epidermal knock down of Basigin had no effect on epithelial cell shape or average cell size at the third instar stage (Figures 7A,B). Moreover, expression and localization patterns of epidermal markers such as coracle, βPS integrin and dE-cadherin appeared unaffected by knock down of Basigin (Figures 7A,C). Class IV dendrites were visualized by tdGFP expressed under the control of the *ppk* promoter (*ppk-CD4tdGFP*) (Han et al., 2011) in *871-Gal4; bsg^+/–^; UAS-bsgRNAi* background. Epidermis-specific knock down of Basigin in this manner resulted in aberrant morphogenesis of neuronal dendrites. Compared to class IV neurons in control larvae, those in larvae expressing RNAi transgenes against Basigin had significantly lower dendrite coverage at 3^*rd*^ instar (Coverage density, Control: 61.80 ± 3.06, *N* = 16; Bsg-RNAi: 55.75 ± 2.64, *N* = 15; *p* = 0.017; Figures 7D,E). However, we did not detect a significant change in dendrite branching at the same stage (No. of nodes per quadrant: Control: 244.06 ± 58.97, *N* = 17 and Bsg-RNAi: 224.77 ± 62.20, *N* = 13, *p = 0.398*.) Epidermal knock down of Basigin had no effect on class I ddaE dendrite length, similar to the lack of effect of *bsg* mutation in class I neurons (Control: 1337.78 ± 181.82 mm, *N* = 8; Bsg-RNAi: 1225.58 ± 176.84 mm, *N* = 8, *p* = 0.231; Figures 7F,G). Likewise, no change was observed in branching of class I neurons (Control: 16.5 ± 2.78, *N* = 8; Bsg-RNAi: 18.75 ± 2.19, *N* = 8, *p* = 0.095; Figures 7F,H). Thus, in addition to cell autonomous roles in class IV neurons, our results suggest cell non-autonomous roles for epidermal-derived Basigin in dendrite morphogenesis.

## Discussion

Dendrite development is controlled by a diverse array of cell surface proteins that together provide information about the neuron’s cellular and molecular milieu. We took an expression pattern-based approach using GFP trap lines to identify candidate regulators of dendrite morphogenesis in the *Drosophila* larval PNS. In follow up experiments, we found that the immunoglobulin superfamily member Basigin is important for formation of complex dendritic arbors. We propose that Basigin mediates interactions with nearby epidermal cells. Our data reveal new insight into roles for the conserved small IgSF molecule in neuronal morphogenesis and point to a pathway from substrate interactions to cytoskeleton in dendritic patterning.

### Screening GFP Trap Lines to Identify Factors Involved in Dendritic Morphogenesis

Large-scale GFP trap collections have been instrumental in identifying proteins that are at the right place to be involved in many different cellular processes, and can complement insights gained from forward genetic screens (Morin et al., 2001; Buszczak et al., 2007; Quiñones-Coello et al., 2007). By screening pre-existing GFP trap lines from three collections we identified diverse candidate regulators of dendrite morphogenesis that can be followed up systematically using mutant analysis, including transcription factors, cell adhesion molecules, cytoskeletal regulators, and signaling proteins. One advantage of a protein expression pattern-based approach is that follow up can be hypothesis-driven, since candidates can be picked based on biological processes or putative molecular function. Indeed, we previously showed that one candidate from this screen, the Scalloped transcription factor, acts at the top of a repressive transcriptional cascade to diversify sensory neuron morphology (Corty et al., 2016). Here we followed up on Basigin-GFP, which showed expression in dendrites and substrate epidermal cells, to study factors that could be involved in dendrite-substrate interactions. Interactions between dendrites and their extracellular environment are increasingly recognized as a central driving force in dendrite morphogenesis and it will be important to further examine how Basigin fits into the multitude of cues that have been identified so far (Parrish et al., 2009; Han et al., 2012; Kim et al., 2012; Dong et al., 2013; Salzberg et al., 2013; Jiang et al., 2014; Cherra and Jin, 2016; Díaz-Balzac et al., 2016; Meltzer et al., 2016; Tenenbaum et al., 2017; Poe et al., 2017; Hoyer et al., 2018).

### Role of Basigin in Dendrite Morphogenesis

Our MARCM analysis of Basigin revealed a decrease in dendrite coverage, dendrite branching, and total dendrite length of Basigin-deficient class IV sensory neurons. Basigin has been implicated in diverse biological processes in vertebrates and invertebrates from embryonic membrane apposition (Reed et al., 2004), embryo implantation (Igakura et al., 1998), tumor invasion (Muramatsu and Miyauchi, 2003), synapse formation (Besse et al., 2007), to cell surface localization of lactate transporters (Kirk et al., 2000). A common theme spanning known functions of Basigin is mediation of cell-cell or cell-substrate interaction, which are critical processes for tissue development and integrity. Such a role is also well-suited to mediate neuronal growth over both small and large spatial domains to achieve precise innervations. Our Basigin loss of function analysis fits with this perspective.

Our experiments suggest that Basigin function in dendrites involves engagement of its extracellular Ig domains by extrinsic effectors, which may be molecules residing on, or released from, epithelial cell surfaces. Non-neuronal Basigin may be one such extracellular effector, since knockdown of Basigin in epidermis also led to defects in class IV dendrite morphogenesis (Alizzi et al., 2020; this study). Similarly, at the fly NMJ, Basigin is required both in the postsynaptic muscles and presynaptic motor neurons for synapse development and function (Besse et al., 2007). Electron micrographs of the larval body wall show that da neuron dendrites are in apposition to epithelial cell surfaces (Han et al., 2012; Kim et al., 2012). Interestingly, *Drosophila* Basigin has been reported to cause cell aggregation when expressed in S2 cells suggesting a homophilic binding capacity (Besse et al., 2007). Isoform-specific homophilic binding has also been reported for vertebrate Basigin (Hanna et al., 2003). Likewise, we propose that Basigin mediates dendrite-substrate interactions to promote complex dendrite morphogenesis. Understanding the factors that regulate the expression and localization of Basigin will be important future directions. The *bsg^–/–^* phenotypes that we observed were consistent with a report that identified Basigin as a target of the RNA binding protein Found in neurons (Fne) in the control of space-filling dendrite growth of class IV neurons, which provides insight into the regulation of Basigin expression (Alizzi et al., 2020). Our data also indicate that the localization of Basigin aligns well with the septate junction protein Coracle and so it could conceivably be a component of junctional complexes.

### Mechanism for Cell Autonomous Role of Basigin in Dendrites

How might Basigin function to promote dendrite patterning? In one scenario, interactions with the epidermis may coordinate addition of new branches throughout the dendritic arbor, thereby maintaining the arbor’s space-filling property. Signals derived from the epidermis regulate scaling growth of dendritic territories as the body wall expands during larval growth (Parrish et al., 2009). The phenotypes we observed in *bsg^–/–^* class IV neurons are unlikely to reflect defects in scaling of overall dendritic territory, as the dendritic field continues to expand in *bsg^–/–^* neurons with no net difference in dendritic area compared to control neurons. Instead, our results may point to an “intra-arbor scaling” process involving Basigin that coordinates dendrite coverage density with the growth of the substrate. In this model, lack of Basigin impairs space-filling growth within the dendritic tree, which normally serves to maintain coverage over the expanding body wall. Comparison between 2^*nd*^ and 3^*rd*^ instar revealed that the failure results from inadequacy, rather than inability, of branch addition through these stages. It remains unclear, however, whether primary and higher order branches are differentially affected. Future studies employing live imaging approaches would be well positioned to offer a nuanced understanding of space filling defects with branch order resolution in Bsg mutants.

The mechanism by which Basigin promotes dendrite coverage likely involves the positively charged KRR motif in the intracellular tail given the necessity of this region for rescue of the Basigin mutant phenotype. Although the molecules that bind to this motif in Basigin are unknown, evidence from studies on other transmembrane proteins identifies the KRR motif as a binding site for cytoskeletal regulators, specifically those of the Ezin/Radixin/Moesin (ERM) family (Yonemura et al., 1998). Therefore, Basigin may impact the neuronal cytoskeleton via regulators that bind to the KRR motif. This assertion is consistent with prior results showing that Bsg is important for stabilization of the neuronal cytoskeleton (Alizzi et al., 2020). Although the effects exerted by such a mechanism are likely to be local, our findings do not rule out other pathways that result in more global effects over the entire dendritic tree. For example, vertebrate Basigin is known to induce global cellular changes such as activation of signaling pathways (e.g., ERK1/2 signaling) and gene expression (Belton et al., 2008).

### Ig Superfamily Members in Neuronal Morphogenesis

Proteins of the Ig superfamily are implicated in nearly all aspects of neural circuit development, including axon growth (Usardi et al., 2016), dendrite targeting (Yamagata and Sanes, 2012) and synapse specificity (Carrillo et al., 2015). Ig superfamily proteins have diverse structures with varying number of Ig domains with or without other identifiable functional motifs. In mice, there are two Basigin isoforms with two and three Ig domains each (Muramatsu, 2016), while humans have two additional isoforms each with a lone extracellular Ig domain (Liao et al., 2011). In *Drosophila*, Basigin is a 2-Ig protein (Besse et al., 2007), but a third Ig domain is also predicted in the longest isoform (B. Shrestha, personal observation). Thus, Basigin is structurally similar to the small IgSF protein family, of which the 2-Ig domain family is best known and implicated in various aspects of neural development. The latter includes the Beat proteins, involved in axon guidance, in *Drosophila* (Pipes et al., 2001), and the ZIG proteins in *C. elegans*, some of which are important for axon and synapse maintenance (Howell and Hobert, 2016). Notably, and potentially analogous to the role of Basigin in dendrite development, ZIG-10 in *C. elegans* is expressed in the epidermis and the motor neurons that physically contact them (Cherra and Jin, 2016) and loss of ZIG-10 in either cell decreases synapse number. Additionally, this function of ZIG-10 requires membrane tethering. Thus, our findings linking *Drosophila* Basigin to dendrite development contribute to a growing body of literature implicating small 2- or 3-Ig containing IgSF members in neuronal morphogenesis.

It will be important to further dissect roles for other small Ig proteins in dendrite development and to extend these analyses to vertebrate systems. Vertebrate Basigin is most closely related to Embigin and Neuroplastin, and the family comprising these three proteins collectively mediate processes ranging from tumor metastasis to embryo implantation and synapse formation (Muramatsu, 2016). At the molecular level, these proteins have diverse functions: as chaperones for monocarboxylate transporters, aiding their cell-surface localization (Kirk et al., 2000), as inducers of MMP expression (Toole, 2003), organizers of cellular cytoskeleton (Curtin et al., 2005; Besse et al., 2007), and as auxiliary subunits of plasma membrane Ca^2+^-ATPases (Schmidt et al., 2017). Embigin induces motor nerve terminal sprouting at vertebrate NMJs (Lain et al., 2009), while Neuroplastin is important for synapse development and function (Sarto-Jackson et al., 2012; Herrera-Molin et al., 2014; Carrott et al., 2016). Basigin itself exhibits broad CNS expression in both mice and humans [Allen Mouse Brain Atlas, Allen Human Brain Atlas (Lein et al., 2007; Hawrylycz et al., 2012)]. Basigin has been shown to regulate the expression and surface localization of monocarboxylate transporters in retinal pigment epithelia in mice, and mice lacking Basigin exhibit degeneration of photoreceptors and are blind (Philp et al., 2003). Our findings in *Drosophila* that Basigin functions in dendrite morphogenesis raise the possibility that it plays similar roles in development of the vertebrate nervous system.

## Data Availability Statement

The raw data supporting the conclusions of this article will be made available by the authors, without undue reservation.

## Author Contributions

BS and WG conceived and designed the study and wrote the manuscript. BS and AB performed experiments, collected data, and analyzed results. WG supervised the project. All authors contributed to the article and approved the submitted version.

## Conflict of Interest

The authors declare that the research was conducted in the absence of any commercial or financial relationships that could be construed as a potential conflict of interest.

## Publisher’s Note

All claims expressed in this article are solely those of the authors and do not necessarily represent those of their affiliated organizations, or those of the publisher, the editors and the reviewers. Any product that may be evaluated in this article, or claim that may be made by its manufacturer, is not guaranteed or endorsed by the publisher.
