# Supplementary material for: The Immunoglobulin Superfamily Member Basigin Is Required for Complex Dendrite Formation in Drosophila
Source: Front Cell Neurosci. 2021 Nov 4;15:739741. doi: 10.3389/fncel.2021.739741 (PMC8600269; doi:10.3389/fncel.2021.739741)
Supplement: Supplementary file 1 [file Data_Sheet_1.docx]

**
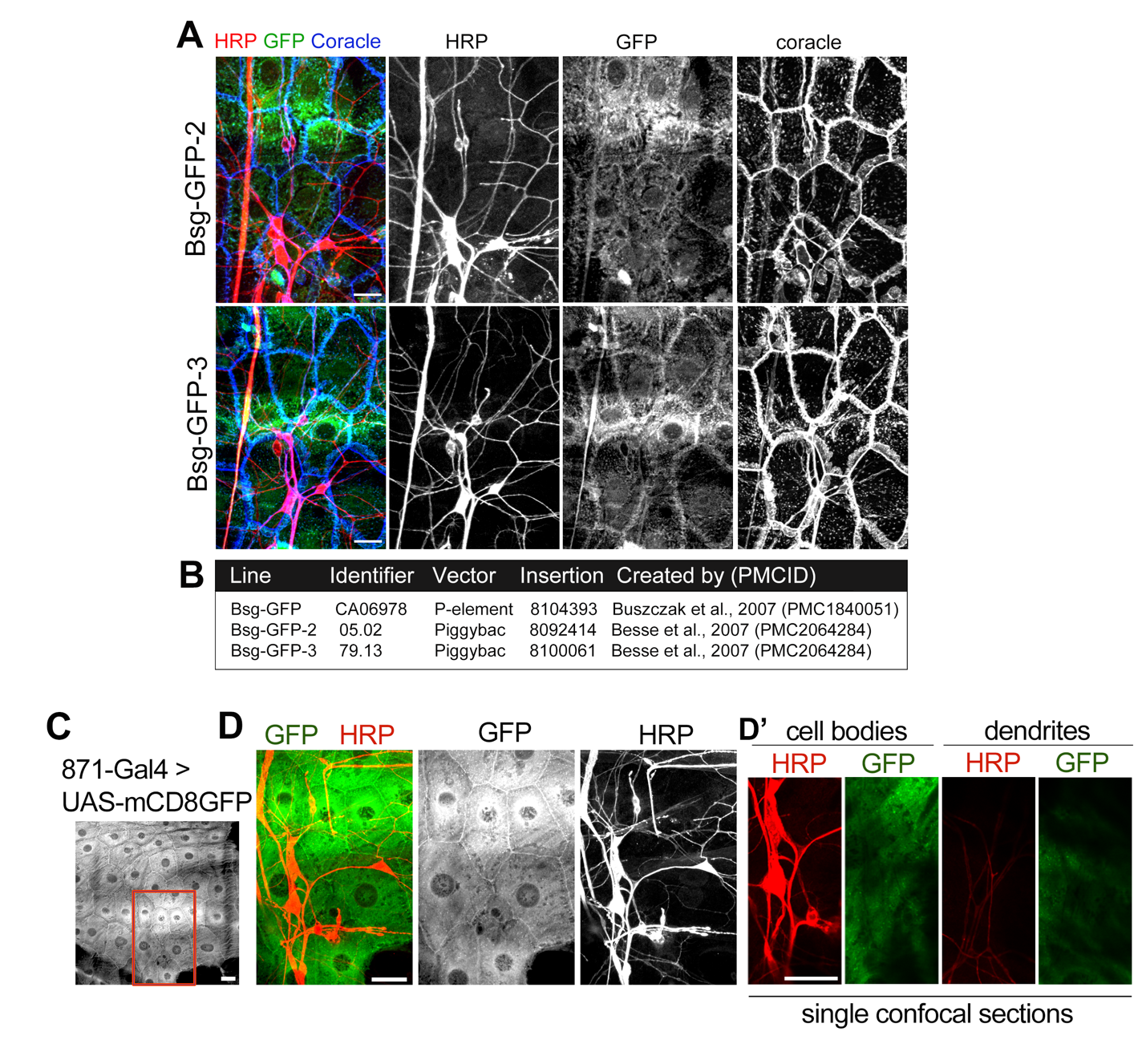
Supplemental Figure 1**

**(A)** Basigin::GFP expression in the *Drosophila* larval body wall at third instar stage in additional GFP-trap lines. Bsg-GFP-2 and Bsg-GFP-3 exhibit similar Basigin expression and localization patterns as those observed in Bsg-GFP (shown in Figure 3A) despite differences in placement of GFP-coding exon. **(B)** Information highlighting differences among the three Basigin::GFP trap lines examined in this study. **(C,D)** 871-Gal4 drives expression of the fluorescent reporter UAS-mCD8GFP in epidermal cells but not da sensory neurons. Panels in **(D)** show close-up of the region outlined in **(C)**. GFP was not detected in da neurons even when single confocal sections of cell body and dendrite-rich regions of the body wall, identified based on HRP signal, **(D’)** were scrutinized.

**
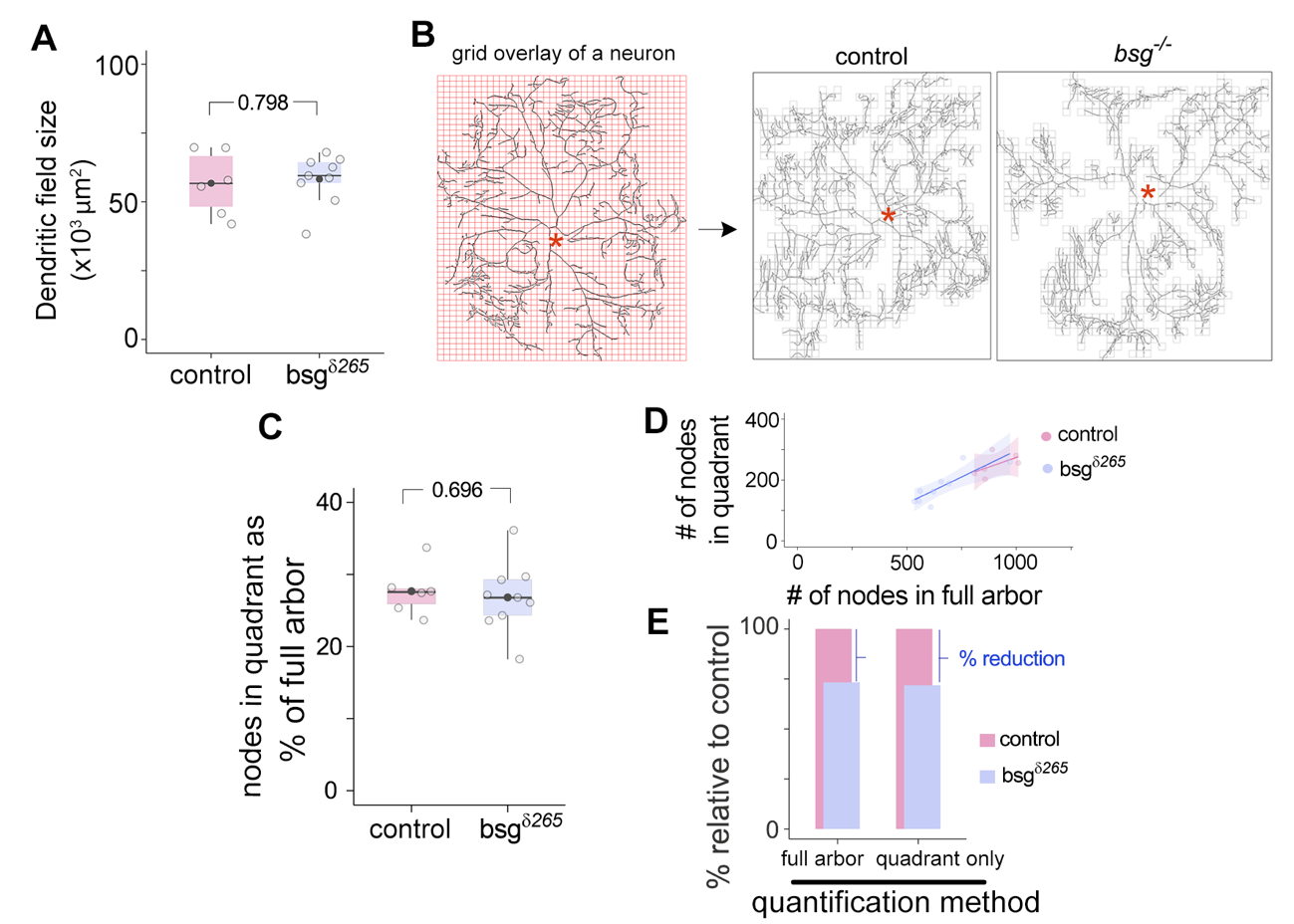
Supplemental Figure 2**

**(A)** No difference was found in the dendritic field sizes of control and *bsg^-/-^*  class IV neurons. **(B)** Quantification of coverage index. A traced or raw confocal image of class IV dendritic tree was overlaid with a grid of square boxes and the number of boxes containing dendrites was counted. In the example shown, a traced class IV arbor is overlaid with a grid, and boxes containing dendrite are automatically detected using the Box Counting feature in FracLac plugin for ImageJ. Red asterisks mark cell body position. See methods for more detail. In some lines of experiments, analysis of class IV (ddaC) neuron was focused on branches in the posterodorsal quadrant. **(C)** The number of nodes in the quadrant comprised equivalent proportion of that over the full arbor in both control and *bsg^-/-^*  neurons. **(D)** Scatterplot showing relationship between number of nodes in quadrants vs. full arbors. Linear models (solid line) applied separately to the two genotypes revealed similar slopes; shaded regions mark 95% confidence interval for predictions from those linear models. **(E)** The decrease in branching in *bsg^-/-^* neurons constituted equivalent proportions (annotated as ‘% reduction’) of the control neurons when quantified over the full arbor (26.71%) or only in the posterodorsal quadrant (28.17%). *p* values are indicated for Welch’s t-test.
